# Proximal Interphalangeal Joint Arthroplasty Using a Silicone Implant: A Comparison Between Integra and NeuFlex in 72 Cases

**DOI:** 10.1177/15589447221122829

**Published:** 2022-09-27

**Authors:** Kelly Weistra, Hester J. Kan, Veerle A. H. J. van Alebeek, Marco J. P. F. Ritt

**Affiliations:** 1 Amsterdam UMC and University of Amsterdam, The Netherlands; 2 The Hand Clinic, Amsterdam, The Netherlands

**Keywords:** proximal interphalangeal joint arthroplasty, NeuFlex, Integra, silicone or silastic PIP joint implant, osteoarthritis, hand surgery

## Abstract

**Background:**

Osteoarthritis of the hand can lead to pain, stiffness, and deformation, and thus to functional disability. The purpose of this study was to compare short-term clinical outcomes of 2 silicone proximal interphalangeal (PIP) joint implants, NeuFlex and Integra, in patients with primary osteoarthritis.

**Methods:**

We included 72 PIP joints, of which 40 were replaced by a NeuFlex implant and 32 by an Integra implant. The average follow-up was 12 months for the Integra group and 16 months for the NeuFlex group.

**Results:**

There was no change in active flexion preoperatively and postoperatively. Extension lag and Disabilities of the Arm, Shoulder, and Hand score decreased substantially in both groups, whereas grip strength and Patient-Specific Functional Scale (PSFS) score increased. All patients were satisfied. Between groups, there was a significant difference in the PSFS score, in favor of the Integra group.

**Conclusions:**

Both implants have excellent results, but more research is needed with more patients to prevent bias and to determine the long-term outcome of these implants.

## Introduction

Osteoarthritis of the hand can lead to pain, stiffness, and deformation, and thus to functional disability. Patients who have osteoarthritis have impaired quality of life (QoL) as studied by Michon et al.^1^ Conservative treatments are helpful in reducing symptoms such as pain, swelling, and stiffness in the onset of osteoarthritis.^2-4^ With progression of symptoms, surgical treatments such as joint arthroplasty become an option.

Most of the attention in the literature focuses on the proximal interphalangeal (PIP) joint. In particular, current research on the subject has been restricted to comparisons of surgical approaches such as the dorsal (Chamay or Swanson) approach, the lateral (Merle) approach,^5^ or the volar (Simmen) approach.^6,7^ Other studies have compared the quality and outcome of PIP implants made from different materials.^6,8^ Literature comparing PIP implants of the same material is scarce.^9^ Most of the studies consist of case series of a single implant, and for silicone PIP implants, they have comparable outcomes when it comes to function and patient satisfaction.^3^

In our institution, NeuFlex (Johnson & Johnson, New Brunswick, New Jersey) was used till mid-July 2019 when the manufacturer quite suddenly stopped production and distribution of this implant in the Netherlands. Our clinic consequently had to switch to the Integra implant (Smith & Nephew, London, UK). The purpose of this single-institution cohort study was to investigate the outcome of these two silicone PIP implants, with a follow-up time ranging from 6 months to 101 months.

## Methods

### Patients

This study analyzes data from two cohorts. The first cohort includes all patients who received a NeuFlex silicone PIP joint implant from March 2013 till mid-July 2019. The second cohort includes all patients who received an Integra silicone PIP joint implant from mid-July 2019 till March 2021.

All patients who received a PIP joint arthroplasty are documented in a single-center registry database by the product name of the used PIP implant. Inclusion criteria were patients aged 18 years or older at the time of implant who received one or more primary PIP implants due to osteoarthritis requiring arthroplasty, with at least 6 months of follow-up. Patients requiring arthroplasty because of rheumatoid arthritis were excluded.

Patients were routinely assessed preoperatively and 1 year after surgery. When the postoperative appointment at that time could not take place due to the coronavirus pandemic or other reasons, patients came back somewhat later for their follow-up visit. See Table 1 for patient characteristics.

**Table 1.** Patient Characteristics by Implant Type.

Patient characteristics/implant type	Integra	NeuFlex
	n = 30	n = 42
Sex, No. (%)		
Female	26 (86.7)	38 (90.5)
Male	4 (13.3)	4 (9.5)
Mean age, y	68.6 (SD = 6.0)	60.9 (SD = 10.8)
Diagnosis, No. (%)		
Osteoarthritis	28 (93.3)	36 (85.7)
Posttraumatic arthritis	2 (6.7)	6 (14.3)
Site of onset, No. (%)		
Left	12 (40.0)	16 (38.1)
Right	18 (60.0)	26 (61.9)
Dominant hand affected	13 (43.3)	29 (69.0)
Median follow-up time, mo Range follow-up time, mo	12 (SD = 9.3) 6-36	16 (SD = 27.8) 9-101

### PIP Implant Surgery and Rehabilitation Protocol

All arthroplasties were performed by a total of 10 well-experienced hand surgeons. All PIP implants were inserted using the dorsal tendon splitting approach (Swanson). Each patient followed the same standard rehabilitation protocol. This encompasses to start exercising actively, three to five days after surgery. A booklet with specific exercises is provided and discussed by a hand therapist during the preoperative intake. During this first postoperative visit, a thermoplast splint with extension block and allowing only 30° of flexion motion is fitted. Patients need to wear this splint day and night. The freedom toward flexion motion is increased each week with 10°. The splint needs to be worn until six weeks after surgery. In weeks 6 to 12 following surgery, there are weekly appointments with the hand therapist to regain mobility, strength, and stability. Full strength and return to normal daily activities are to be expected at around three months postoperatively.

### Outcome Measures

Primary outcomes were function, measured by active range of motion (AROM) in degrees, extension lag in degrees, and grip strength in kilogram-force tested using a JAMAR dynamometer (Sammons Preston, Bolingbrook, Illinois). Other primary outcomes were pain evaluated with Visual Analog Scale (VAS) scores (scale 0-100, with 0 indicating no pain and 100 indicating intolerable pain), disability measured with the Disabilities of the Arm, Shoulder, and Hand (DASH) questionnaire (scale 0-100, with 0 indicating no disability at all; a normal, average person scores between 10 and 15), and patient satisfaction evaluated using the Patient-Specific Functional Scale (PSFS; scale 0-10, with 0 being unable to perform the activities and 10 being able to perform the activities at the same level as before the injury or problem). Patients were asked to fill in the DASH and PSFS online prior to the follow-up meeting. If they did not succeed in filling these forms, the hand therapist assessed those forms orally during the appointment. In addition, patients also had to grade three questions about satisfaction (scale 1-10, 1 being not satisfied at all and 10 being fully satisfied): “Are you satisfied with the result?,” “Did the surgery lead to an improvement?,” and “Would you do the surgery again?” Secondary outcomes were complications/revisions and implant survival.

### Statistics

Patients were allocated to one of the two groups based on the PIP joint implant used. Descriptive statistics for patient characteristics are presented as frequencies and percentages. For age, AROM, extension lag, and revision rates, medians or means and standard deviations were calculated. We chose to divide AROM into a flexion and extension trajectory. The significance level was set at *P* < .05. We chose to perform unpaired *t* test and used the Mann-Whitney *U* test for differences between groups, depending on the distribution of the outcome variables. The paired *t* test and Wilcoxon signed rank test were conducted for differences between baseline and follow-up. We performed statistics on AROM, extension lag, grip strength, VAS, DASH, PSFS, and satisfaction about surgery.

## Results

There were huge improvements in all outcome measures for both groups when comparing baseline and follow-up (*P* = .002 to *P* < .001), except for flexion (*P* = .653). Table 2 provides an overview of all the outcomes with the corresponding *P* values.

**Table 2.** Difference Between Baseline and Follow-up Measured for All Patients by Outcome Measures.

Timeline/parameters	Flexion,^a^ deg	Extension lag,^a^ deg	Grip strength,^b^ kgf	VAS score^a^ (0-100 )	DASH score^a^ (0-100)	PSFS^b^ (0-10)
	Median (SD)	Median (SD)	Mean (SD)	Median (SD)	Median (SD)	Mean (SD)
Baseline	71.0 (19.7)	14.5 (10.8)	14.8 (6.5)	75.0 (16.2)	38.8 (16.8)	3.6 (1.7)
Follow-up	75.0 (21.6)	6.5 (11.5)	16.9 (5.9)	0.0 (25.8)	16.1 (14.4)	6.5 (2.2)
*P* value	.653	<.001*	.002*	<.001*	<.001*	<.001*

*Note.* VAS = Visual Analog Scale; DASH = Disabilities of the Arm, Shoulder, and Hand; PSFS = Patient-Specific Functional Scale.

* Difference is significant.

a Statistics by Wilcoxon signed rank test.

b Statistics by paired *t* test.

In Table 3, medians, means, and statistical analyses of the outcome variables are described. Considering all of the outcome measures, we see that there is no statistically significant difference between the two groups, except for the PSFS. With unpaired *t* test, it gives a difference of means of 1.3653 (95% confidence interval, 0.271-2.7035, *P* = .046). There was no significant difference when comparing the baseline values of the NeuFlex and Integra group (*P* = .124).

**Table 3.** Statistical Analyses of the Difference in Baseline and Follow-up Between Integra and NeuFlex by Outcome Variable.

Timeline/means of parameters	Flexion,^a^ deg	Extension lag,^a^ deg	Grip strength,^b^ kgf	VAS score^a^ (0-100)	DASH score^a^ (0-100)	PSFS^b^ (0-10)	Satisfaction with surgery^a^ (0-10)
	Median (SD)	Median (SD)	Mean (SD)	Median (SD)	Median (SD)	Mean (SD)	Median (SD)
Integra							
Baseline	72.5 (17.0)	15.0 (10.6)	14.3 (7.0)	80.0 (13.9)	40.8 (19.0)	3.2 (2.0)	
Follow-up	80.0 (22.1)	10.0 (13.2)	17.6 (6.7)	0.0 (26.6)	7.1 (14.2)	6.9 (2.3)	9.0 (2.8)
Difference^c^	−4.0 (25.3)	5.0 (17.0)	−3.3 (4.6)	62.5 (28.0)	24.1 (20.1)	−3.7 (2.7)	
NeuFlex							
Baseline	70.0 (21.3)	13.0 (11.8)	15.2 (6.3)	75.0 (17.7)	38.2 (15.4)	3.9 (1.4)	
Follow-up	70.5 (20.9)	0.0 (9.9)	16.5 (5.3)	0.0 (25.3)	18.6 (14.6)	6.2 (2.1)	8.0 (2.1)
Difference^c^	1.5 (−2.0)	10.5 (13.3)	−1.3 (4.4)	70 (29.8)	19.3 (12.8)	−2.3 (2.6)	
*P* value	.489	.290	.130	.907	.179	.046*	.268

*Note.* VAS = Visual Analog Scale; DASH = Disabilities of the Arm, Shoulder, and Hand; PSFS = Patient-Specific Functional Scale.

* Difference is significant.

a Statistics by Mann-Whitney *U* test.

b Statistics by unpaired *t* test.

c Difference between baseline and follow-up.

Our secondary outcome measures were complications, revisions, and implant survival. There were no infections and five revisions during the 1-year follow-up period. Three cases were revisions in the NeuFlex group: twice an arthrolysis was performed and once an arthrolysis plus tenolysis. Two cases were noted in the Integra group: one needed arthrolysis, and in one case, an implant switch was performed, from Integra to NeuFlex, because of pain. No implant breakage was observed during the 1-year follow-up.

## Discussion

Replacing a finger joint by an implant has very satisfying results. The main indication to perform a finger joint arthroplasty is to decrease pain. On average, the (already limited) range of motion will be maintained, the extension lag can decrease somewhat, and in general patient satisfaction is high.^6,10^ The QoL increases after surgery.^11^ To date, silicone implants provide consistent and actually better results in comparison with implants of other materials such as the polyethylene-metal or pyrocarbon.^6^ Other materials for small joint implants have been tried but mostly discarded due to inferior results to silicone,^12^ which now have been widely used in a clinical setting since 1968. Moreover, although implant failure is seen in silicone implants as well, most of the times these hardware failures remain clinically unnoticed and do not always necessarily lead to revision.^13,14^

Research on NeuFlex implants has shown excellent and reliable results.^8,15^ The current literature has proven the success of the NeuFlex implants for metacarpophalangeal joint implants over other implants multiple times.^9,16-19^ For PIP joint implants, there is only one article comparing NeuFlex with Swanson silicone implants (Wright, Staines-upon-Thames, UK). This study showed similar outcomes between the two implants.^20^ The goal of our study was to compare the short-term clinical outcome of two silicone PIP joint implants (NeuFlex and Integra), using a dorsal approach, in patients with primary osteoarthritis. Overall, combining both groups, our study shows good short-term results of this arthroplasty using a silicone implant, with high levels of satisfaction, and our results are comparable to reports in the literature when it comes to pain, DASH, and patient satisfaction.

It is well known that PIP joint arthroplasty could lead to a deficit in flexion or cause an extension lag. Compared with the literature, we have surprisingly good outcomes for AROM (extension lag and flexion) in both groups. This could be a result of our strict and somewhat different rehabilitation protocol or the fact that on average we operated upon relatively supple, but painful joints.

Moreover, it is reasonable to assume that hand strength will decrease after arthroplasty, but this did not occur in our groups. In fact, there was even a slight improvement, probably as a result of the reduction in pain after surgery and the strict rehabilitation protocol. It should be mentioned that these outcomes (extension lag, active flexion, and strength) are highly dependent on the included patient population, inclusion of patients with rheumatoid arthritis would probably give worse outcomes.

Differences in outcome between the two groups (NeuFlex and Integra) were not found, except for a small but significant functional improvement measured using the PSFS questionnaire. This lack of differences could have several causes. First, although compared with the literature we conducted a study including a relatively large number of implants, the study size could still be too small to find statistical significant differences. Second, we report only on short-term results (median = 12-16 months; range = 6-101 months), and results after a longer follow-up time might be different. Third, not all relevant data were available. For example, there is no information on bone stock, soft tissue quality, and lateral stability. As mentioned above, there was a significant difference in the PSFS findings in favor of the Integra group. When looking at the preoperative baseline measures of both implant groups, there is no significant difference; thus, the difference between the outcomes in these two groups is a valid finding.

One of the strengths of our study is that we excluded patients with rheumatoid arthritis to maintain homogeneity of our data set. It is known from the literature that patients with rheumatoid arthritis have less satisfying results after small joint arthroplasty compared with patients with primary osteoarthritis, and the reason for this is multifactorial.^14^ Nonetheless, this patient group is also very important for the overall results of silicone arthroplasty but should be analyzed and judged separately. Some of the articles in the literature did their analyses on patients with rheumatoid arthritis or arthritis after injury exclusively.^6,21^ Comparison between rheumatoid and nonrheumatoid patient groups should be made with caution in this regard. It is quite possible that this at least partially explains the lower outcomes in range of motion and the higher outcomes in postoperative pain scores of these articles. Second, the fact that all patients followed the same strict and refined rehabilitation protocol is to be considered a strength of our study and could be responsible for the good results in AROM. Further research is needed to study the differences and outcomes of this rehabilitation protocol in comparison with other existing protocols.

Challenges for this study are the number of patients to carry out statistical analyses. Selection bias could more easily occur with small numbers of patients in a study because they are not a good reflection for the entire patient population. Regarding the questionnaires, it would be better if they were taken by one and the same hand therapist, so observer bias due to interpretation errors could be prevented. In addition, with the inclusion of more outcome variables such as bone stock and lateral stability and with a more suitable measurement instrument specifically for fingers, like the Michigan Hand Questionnaire (MHQ), possible differences in performance of the two spacers could come to light. Research from Hensler et al^22^ shows that patients with silicone implants tend to have more lateral instability than patients with surface replacement implants. Although it does not seem to pose a clinical problem, research toward lateral stability between groups of silicone implants has not been published yet in literature. Finally, patients were operated by 10 surgeons with different experience and surgical technique, albeit using the same dorsal approach and working in one clinic and using the same protocol. This could be regarded as a disadvantage of our study, but, on the other hand, it could also be seen as an advantage because it provides a realistic reflection of the average clinical results at a certain institution.

## Conclusion

Overall, we can conclude that PIP silicone implant arthroplasty has excellent results in patients with painful primary osteoarthritis. We could not demonstrate any differences between NeuFlex and Integra implants, except for the PSFS. Nonetheless, more research should be done with greater patient groups so that potential bias is prevented and to determine the long-term results of these implants.
